# Parental praise and children’s exploration: a virtual reality experiment

**DOI:** 10.1038/s41598-022-08226-9

**Published:** 2022-03-23

**Authors:** Eddie Brummelman, Stathis Grapsas, Katinka van der Kooij

**Affiliations:** 1 Research Institute of Child Development and Education, University of Amsterdam, P.O. Box 15780, 1001 NG Amsterdam, The Netherlands; 2 Utrecht University, Utrecht, The Netherlands; 3 Vrije Universiteit Amsterdam, Amsterdam, The Netherlands

**Keywords:** Human behaviour, Motor control

## Abstract

When children practice a new skill and fail, it is critical for them to explore new strategies to succeed. How can parents encourage children’s exploration? Bridging insights from developmental psychology and the neuroscience of motor control, we examined the effects of parental praise on children’s motor exploration. We theorize that modest praise can spark exploration. Unlike inflated praise, modest praise acknowledges children’s performance, without setting a high standard for future performance. This may be reassuring to children with lower levels of self-esteem, who often doubt their ability. We conducted a novel virtual-reality experiment. Children (*N* = 202, ages 8–12) reported self-esteem and performed a virtual-reality 3D trajectory-matching task, with success/failure feedback after each trial. Children received modest praise (“You did well!”), inflated praise (“You did *incredibly* well!”), or no praise from their parent. We measured motor exploration as children’s tendency to vary their movements following failure. Relative to no praise, modest praise—unlike inflated praise—encouraged exploration in children with lower levels of self-esteem. By contrast, modest praise discouraged exploration in children with higher levels of self-esteem. Effects were small yet robust. This experiment demonstrates that modest praise can spark exploration in children with lower levels of self-esteem.

## Introduction

Like other children her age, Sally is intrinsically curious and often spontaneously practices new skills. Today, her teacher encourages her to draw a house with a chimney. But when she shows the teacher her drawing, the teacher is not happy and asks her to try again. She picks up a blank piece of paper and tries the same way of drawing the house, without any luck. It is not until she tries a different drawing strategy that she succeeds. Such failure-induced variability in strategy use is known as *motor exploration*, which is central to motor learning^1–3^. Indeed, when children practice a new skill and fail, it is critical for them to explore other strategies to yield success^2,3^. Motor exploration is a new and burgeoning area of research^4^. Scholars have begun to study the determinants of motor exploration in children^5^. To date, little is known about how parents can encourage children’s motor exploration. We conducted a novel virtual reality experiment to examine the effects of parental praise on children’s motor exploration. Doing so, we bridged a developmental-psychological perspective on social feedback^6–8^ with a neuroscience perspective on motor exploration^9–11^, while advancing research on how parents can encourage children’s exploration^12^.

Since the 1970s, Western parents have embraced the practice of praising children^7,13^. Because exploration is risky (as it might not yield success immediately), it seems quite intuitive that saying positive things about children will give them the self-confidence they need to explore. Indeed, parents spontaneously provide praise before and during children’s exploration^12,14^. While parents are right that their words matter, their ideas about how to instill self-confidence may sometimes be misguided^15,16^. Research has begun to reveal that certain types of praise can have detrimental effects on children’s self-confidence and motivation^17,18^. Parents often provide children with inflated praise. About 25% of all praise is inflated^19,20^. Instead of telling children that they did well, parents tell them they did *incredibly* well. Instead of telling children that they a good job, parents tell them they did an *amazing* job. Despite being well-intentioned, inflated praise may not encourage children’s exploration. When children are told they did incredibly well, they may infer that they are expected to do incredibly well all the time^13,19^. As such, inflated praise may be perceived to contain an implicit demand for continued exceptional performance^21–23^. Thus, when children receive inflated praise, they may not feel encouraged to explore new strategies to succeed.

Although inflated praise might not boost exploration, modest praise might. Modest praise contains a positive but modest evaluation (e.g., “You did well!” rather than “You did *incredibly* well!”). Unlike inflated praise, modest praise simply acknowledges and values children’s performance, without demanding continued exceptional performance^19^. This may be especially reassuring to children with lower levels of self-esteem, who often doubt their ability^15,24^. When they perform a challenging task, these children may feel anxious about revealing their low ability^25^ and may worry that they will not be able to maintain their performance^26^. In adults, such a state of anxiety has been shown to reduce exploration^10^. Modest praise may alleviate some of this anxiety. When children with lower levels of self-esteem receive modest praise, they may feel confident that they are able to meet the standards set for them, leading them to explore more. Indirect evidence supports this hypothesis. In one experiment^19^, children made a drawing and then received inflated praise (“You made an *incredibly* beautiful drawing!”), modest praise (“You made a beautiful drawing!”), or no praise from a professional painter. Children with lower levels of self-esteem who received modest praise subsequently embraced more challenging tasks (also see ref^27^). However, the effects of praise on exploration are still unknown. We theorized that, especially to children with lower levels of self-esteem, modest praise may be a reassuring message that encourages exploration.

By studying how social feedback shapes children’s motor exploration, our work builds on a larger literature on exploration in math learning, science learning, and causal reasoning. Although exploration in these domains is driven by higher-level inferential processes than is motor exploration, studying it has provided important insights into the nature of exploration. On the one hand, children learn as they explore the world on their own, such as through exploratory play^28^, discovery learning^29,30^, and trial and error^31^. On the other hand, children learn as they interact with others, such as through collaborative problem solving^32^, direct instruction^8^, and observation of others’ actions^33^. Recent research has shown that these processes are not independent but constitute a dynamic dyadic process^34,35^. For example, children explore more when their parents scaffold their exploratory behaviors^36,37^, engage in well-timed causal talk^12^, and support their autonomy and sense of competence^14,38^. Parents, in turn, engage more in these supportive practices when their children explore. Extending this past work, our work is the first to experimentally isolate the effects of parental praise on children’s motor exploration.

### Present study

We tested our hypothesis in a novel virtual reality experiment that examined motor exploration. We focused on late childhood (ages 8–12), a critical developmental juncture at which children are not only “directed explorers who are hungry for information in their environment”^39^ but also highly sensitive to adults’ evaluations of them and their actions^24,40^. In our study, children first reported their self-esteem and then completed a virtual reality task in which they copied 3D lines using a handheld controller. In the task, children received success or failure feedback on each trial, but they could not see the lines they drew, so they relied on their exploration to yield success—like reaching for a light switch in the dark. Parents participated as naïve experimenters. When children were halfway through the task, parents gave them modest praise (“You did well!”), inflated praise (“You did *incredibly* well!”), or no praise. We measured motor exploration as movement variability following failure. Movement variability following failure indicates that children are varying their movements in search of those that yield success^11,41,42^. We hypothesized that modest praise, relative to no praise, would encourage exploration in children with lower levels of self-esteem. By including an inflated-praise condition, our study was able to disentangle whether the effects were indeed unique to modest praise or shared by different types of praise. We hypothesized that inflated praise would not encourage exploration relative to no praise.

## Methods

### Participants

We report how we determined our sample size, all data exclusions (if any), all manipulations, and all measures in the study. Participants were 202 children (41% girls, 94% Dutch) aged 8–12 years (*M* = 9.69, *SD* = 1.25) and one of their parents (54% female, 92% Dutch) aged 30–57 years (*M* = 41.65, *SD* = 4.87). Participants visited Science Center NEMO, the largest science museum in The Netherlands. This research was part of Science Live, the innovative research program of Science Center NEMO that enables scientists to use NEMO visitors as participants. Because the study ran for two weeks in the science museum, we recruited as many participants as possible during this period. Because our study used virtual reality goggles, children who suffered from epilepsy or wore glasses with diopters greater than − 2 or + 2 could not participate. Parents provided informed consent for their own and their child’s participation; children assented to their own participation. The study was approved by the Scientific and Ethical Review Board (VCWE) of the Faculty of Behavioural and Movement Sciences of the Vrije Universiteit Amsterdam (VCWE-S-19-00082-2) and was performed in accordance with the relevant guidelines and regulations. A video describing our study methods is available on YouTube at: https://www.youtube.com/watch?v=Tku5k7CjP70. Parents provided informed consent for the video to be published in an online open-access publication.

### Procedure

Before the experiment proper, children and parents reported their demographic information, and children completed a trait self-esteem questionnaire. Children completed the questionnaire independently and in silence. Consistent with earlier research^19,27,43^, we administered the questionnaires before (rather than after) the experiment proper to prevent the between-person manipulation of praise from affecting the reports of self-esteem. We also assessed two other personality traits (i.e., narcissism^44^ and social anxiety^45^), which are unrelated to our current research questions and are therefore not reported here. The complete questionnaires and study codebook are available on OSF at: https://osf.io/u3ma8/.

#### Self-esteem

Children completed the Lifespan Self-Esteem Scale^46^, which measures trait self-esteem using four items: “How do you feel about yourself?”, “How do you feel about the kind of person you are?”, “When you think about yourself, how do you feel?”, and “How do you feel about the way you are?” Items were rated on 5-point scales (0 = *Really sad*, 1 = *Sad*, 2 = *Neutral*, 3 = *Happy*, 4 = *Really happy*). Responses were averaged across items (*M* = 3.15, *SD* = 0.56, Cronbach’s *α* = 0.73). Consistent with past research^46^, we analyzed self-esteem as a continuous score, rather than categorizing children as having high or low self-esteem.

#### Virtual reality task

**Figure 1:**
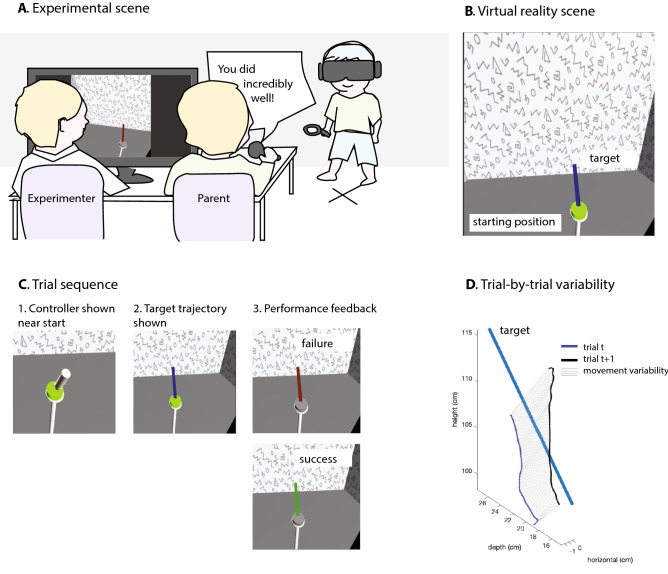
Experimental setup. Panel (**A**): The child performs the 3D trajectory matching task, wearing a virtual reality headset and headphone. The parent sits next to the experimenter, behind the monitor, and is randomly assigned to give modest praise, inflated praise, or no praise through the child’s headphone. Panel (**B**): The task from the child’s perspective. The grey knob turns green when children touch it with the controller. Panel (**C**): An example trial, including visual success and failure feedback. Panel (**D**): An example of how trial-by-trial variability was calculated based on the spatial difference between subsequent movements.

Children then performed a 60-trial virtual reality 3D trajectory matching task that has been developed to index motor exploration as movement variability following failure (Fig. 1, panel A)^47^. Because the science museum imposed a time limit of 20 min per participant, we designed the task to focus exclusively on exploration, without assessing children’s learning, because learning is known to take more time (e.g., in similar tasks, it takes 150–300 trials for learning to plateau)^48–50^. In the task, children wore an HTC Vive virtual reality headset and a headphone. Children viewed a rectangular room with a central pole with a grey knob (Fig. 1, panel B). They were asked to copy a straight 3D line by pulling the handle of the controller and then moving the controller. The grey knob was where children began drawing each trajectory. To make the task equally difficult for children of different heights, the grey knob was set to 80% of children’s height.

Before the task proper, children familiarized themselves with the virtual reality equipment and task in two ways. First, they drew freely, without copying anything. They could see their movements as a pink line that floated in space. Second, they copied a straight 3D line. After doing so once, they completed five practice trials that were identical to the trials in the task proper, except that the lines were displayed in a different angle (to minimize learning effects) and children could see the paths they had drawn after releasing the trigger (to show them how their movements were recorded). After children had completed the practice trials, they were told: “We will now start the 3D task, and you’ll draw a line 60 times. There is a difference [with the practice trials]: you won’t be able to see what you drew anymore. But you will be able to see if you did well. If you did well, the line will turn green. If you didn’t do well, the line will turn red. On the computer we can see what you draw. Your mother [father] can also see, because she [he] will join us in a minute. I will get her [him] now!” When the parent had entered the room, children started the task.

In the task proper, children completed 60 trials in which they copied a straight 3D line as accurately as possible using a handheld controller (Fig. 1, panel C). The line was identical for all children across all trials. Children could *not* see the paths they were drawing, so they had no visual information on how to change the movement to yield success, making them reliant on their exploration. When children pulled the trigger, they initiated the drawing. When children released the trigger, they received either success or failure feedback for 500 ms. After each trial, children returned the controller to the starting position. When the controller approached the starting position (within 3 cm), the controller was displayed as a stylus (with a 1-cm diameter), so that children could return it easily. The next trial began after children returned the controller to the starting position. Each trial began with showing children the target trajectory. A screen capture of the task is available on YouTube at: https://youtu.be/rlIdJZG937o.

To ensure that all children would experience a sufficient and comparable number of failures to measure post-failure movement variability, we used an adaptive success criterion, based on children’s own previous performance (Fig. S1). Here, performance reflects the difference in length and elevation between the target trajectory and the drawn trajectory (i.e., error). When the error was smaller than the median error of their own previous five trials, children received success feedback (i.e., they heard a positive ka-ching sound and the target trajectory was colored green). When the error was larger, children received failure feedback (i.e., they heard a negative buzzer sound and the target trajectory was colored red). On the first trial, all children received failure feedback. On the next four trials, the success criterion was based on the median of the available trials. In previous studies, this adaptive success criterion yielded a success rates of 40–60%^9,49^. In our study, the number of times children received success and failure feedback was distributed equally across experimental conditions (Table S1). In each experimental condition, children received failure feedback on an average of 36 trials (18 pre-manipulation trials, 18 post-manipulation trials), so there were sufficient post-failure trials to examine post-failure movement variability.

#### Parental praise

Unbeknownst to children, and while children were completing the questionnaires and performing the practice trials, parents were instructed about and practiced the praise manipulation in a separate room. We defined praise as positive evaluations of the child’s traits, actions, or products^13,22^. Praise is inflated when it contains an adverb (e.g., *incredibly*) or adjective (e.g., *amazing*) signaling a very positive evaluation^19,20^. Before the child reached the 30^th^ trial, the parent entered the room. On the experimenter’s monitor, the parent watched the child perform the task, including the success and failure feedback the child received. Right after the 30^th^ trial, when the child had a short break, the parent said through the child’s headphone: “Hi [child’s name]. The experimenter and I watched your performance. You are halfway done.” Parents were then randomly assigned to provide modest praise (“You did well!”; *n* = 64), inflated praise (“You did *incredibly* well!”; *n* = 68), or no praise (*n* = 68). All parents then said: “See you later!” Consistent with prior research^27,43^, in both praise conditions, the praise was focused on children’s actions (i.e., doing well on the task, known as process praise). To make sure children heard no sound other than their parent’s words, we played pink noise through their headphones.

#### Perceived sincerity

After the task, children self-reported the perceived sincerity of what their parents said: “During the drawing task your mother/father said something to you through your headphone. What my father/mother said, was…”. They self-reported the perceived sincerity using four items (i.e., *honest, credible, serious, sincere*) on a five-point Likert scale (0 = *Not at all true*, 1 = *Not really true*, 2 = *Not true, not untrue*, 3 = *Sort of true*, 4 = *Completely true*; *M* = 3.06, *SD* = 0.73)^19^. The four items had an internal consistency of Cronbach’s *α* = 0.67.

#### Awareness check

After the task, to examine whether children consciously remembered the feedback their parents had given, children were asked what their parents had said to them through their headphone; children selected one of three options (1 = “You did well!”, 2 = “You did *incredibly* well!”, 3 = neither).

Afterwards, parents and children were thanked and thoroughly debriefed.

### Data preparation

We used a trial-by-trial approach to calculate movement variability in the 3D trajectory matching task (Fig. 1, panel D). We first resampled the drawn trajectory of each trial to 50 positions that were uniformly distributed in space. We then calculated the spatial difference between the trajectory on trial t and the trajectory on trial t + 1 (for average movement variability per task phase and experimental condition, see Table S2). For example, across two subsequent trials, drawing identical lines would result in a variability of zero, but drawing lines that have an identical shape but are centered 3 cm apart would result in a variability of 3 cm.

Before data analysis, we excluded trials with movement artifacts, which we defined stringently as trials in which children’s movement was shorter than 3.78 cm in length (i.e., 3 *SD*s below of the mean log-transformed movement length; 1.92% of total trials). Because movement variability was calculated as the difference in the trajectories between two consecutive trials, we also excluded trials following those with movement artifacts. Ten children could not be included in the analyses because they withdrew from the study (*n* = 3), were interrupted during the task (*n* = 1), had fully missing data on movement variability due to a technical issue (*n* = 5), or had fully missing data on self-esteem (*n* = 1).

Post hoc power analysis with 1,000 Monte Carlo simulations (simr package Version 1.0.5)^51^, based on the effect size obtained in the current study, shows that the final sample size (192 children, yielding 3228 observations) provided acceptable power (72.20%) for detecting the expected modest praise × self-esteem interaction (i.e., -0.369) on exploration (α = 0.05, two-tailed). Despite our directional hypotheses, we used two-tailed testing to provide a conservative test at α = 0.05.

### Data analysis

Our key dependent variable was exploration. Following prior work^11,41,47,48,52^, we operationalized exploration as movement variability following failure (i.e., the difference in drawn trajectory between the post-failure trial and the failure trial). Movement variability following success is distinct from exploration, as it reflects mostly sensorimotor noise (i.e., unintentional deviation from a previous successful movement trajectory). Consistent with the view that movement variability is generally an adaptive response to failure but not success, in the pre-manipulation trials, children showed more movement variability following failure than following success, *B* = 0.39, *SE* = 0.04, *t*(5234) = 10.38, *p* < 0.001, Cohen’s *d* = 0.29.

For our confirmatory analyses, we ran a mixed effects regression model with exploration during the 30 post-manipulation trials as the Level-1 dependent variable. We included pre-manipulation exploration (i.e., average exploration during the 30 pre-manipulation trials), praise as two dummy coded variables (dummy 1: 1 = modest praise, 0 = inflated praise, − 1 = no praise; dummy 2: 1 = inflated praise, 0 = modest praise, − 1 = no praise), self-esteem (mean-centered), and the two-way interactions between the praise variables and self-esteem as Level-2 fixed effects. Dummy 1 compares modest praise to no praise, and dummy 2 compares inflated praise to no praise. We set a random intercept per individual.

If the modest praise × self-esteem interaction was significant, we followed it up using simple slopes analysis (which examines the association between self-esteem and exploration within each experimental condition)^53^ and region of significance analysis (which identifies the exact values of self-esteem at which the effect of modest praise on exploration becomes statistically significant)^54^. In each of these analyses, we treated self-esteem as a continuous score.

Because our study involved a narrow age range, we did not include age in our confirmatory analyses. Age was not significantly correlated with self-esteem, *r*(190) = 0.06, *p* = 0.395, but it was negatively correlated with pre-manipulation exploration, *r*(190) = − 0.27, *p* < 0.001, consistent with the notion that movement variability tends to decrease with age^5^. Including age as a covariate did not change our study findings (Table S3), so we reported our confirmatory analyses without age.

In preliminary and exploratory analyses, we used non-parametric (rather than parametric) tests on the median (rather than mean) of error, because error was skewed and contained outliers; and we conducted Poisson regressions on the number of successes, because success is a count variable.

## Results

### Preliminary analyses

There were no significant condition differences in children’s sex, *χ*^2^ (2, *N* = 192) = 1.32, *p* = 0.516, age, self-esteem, pre-manipulation movement variability following failure (i.e., exploration) or following success, Wilks’ *Λ* = 0.96, *F*(8, 370) = 1.03, *p* = 0.411, or the median of pre-manipulation error, Kruskal–Wallis *H*(2) = 3.11, *p* = 0.211, nor in parents’ sex, *χ*^2^ (2, *N* = 192) = 0.95, *p* = 0.623, or age, *F*(2, 182) = 0.62, *p* = 0.538, which shows that random assignment was successful. In addition, there were no significant condition differences in the frequency of success feedback, neither pre-manipulation, *ps* ≥ 0.828, nor post-manipulation, *ps* ≥ 0.502, which indicates that the adaptive success criterion was successful in distributing feedback equally across conditions. Finally, the awareness check findings show that most children remembered accurately whether and how their parent praised them, *χ*^2^ (4, *N* = 192) = 71.48, *p* < 0.001 (Table S4), which suggests that the experimental manipulation of praise was successful.

### Confirmatory analyses

Results are shown in Table 1. There was a significant main effect of pre-manipulation exploration, *p* < 0.001, such that children who explored more in pre-manipulation trials also tended to explore more in post-manipulation trials. There were no main effects of modest praise, inflated praise, or self-esteem, nor an inflated praise × self-esteem interaction, *ps* ≥ 0.530. Importantly, the predicted modest praise × self-esteem interaction was significant, *p* = 0.012.

**Table 1:** Multilevel analysis examining the effects of praise and self-esteem on children’s exploration.

Fixed effects	*B*	*SE (B)*	*t*
Intercept	1.33	0.11	12.44***
Pre-manipulation exploration	0.45	0.03	15.83***
Modest Praise^a^	0.00	0.08	− 0.05
Inflated Praise^b^	− 0.03	0.08	− 0.43
Self-Esteem	− 0.02	0.10	− 0.17
Modest Praise × Self-Esteem	− 0.37	0.15	− 2.54*
Inflated Praise × Self-Esteem	0.10	0.16	0.63

^a^Modest Praise: 1 = Modest Praise, 0 = Inflated Praise, − 1 = No Praise.

^b^Inflated praise: 1 = Inflated Praise, 0 = Modest Praise, − 1 = No Praise.

*p < .05. **p < .01. *** *p* < .001.

**Figure 2:**
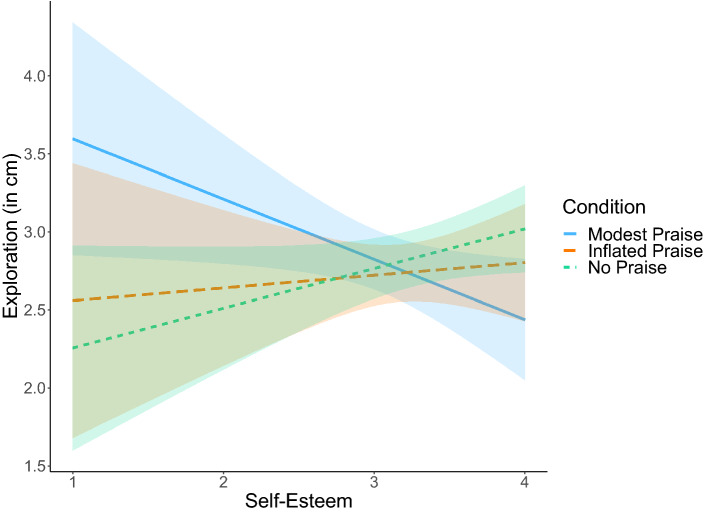
The effects of self-esteem and praise on exploration. The colored regions around the regression lines reflect 95% confidence intervals.

**Figure 3:**
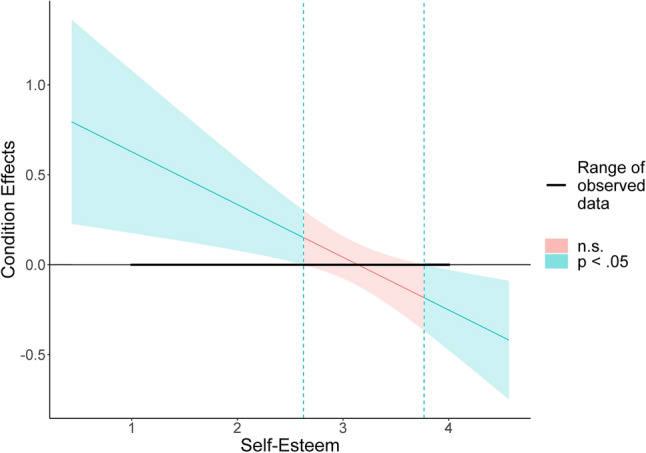
Region of significance of the effects of self-esteem and praise on exploration. The y-axis represents the unstandardized regression coefficient of the condition effect. When self-esteem is outside of the interval (2.63, 3.77), the effect of modest praise, relative to inflated praise and no praise, on exploration becomes statistically significant. In our sample, observed self-esteem scores ranged from 1 to 4.

To follow-up the significant interaction, we first conducted simple slopes analysis (Fig. 2). Self-esteem was not significantly related to exploration after inflated praise, *B* = 0.08, *SE* = 0.20, *t* = 0.40, *p* = 0.689, marginally significantly positively related to exploration after no praise, *B* = 0.25, *SE* = 0.14, *t* = 1.77, *p* = 0.079, and significantly negatively related to exploration after modest praise, *B* = − 0.39, *SE* = 0.18, *t* = − 2.14, *p* = 0.033. We then conducted region of significance analysis (Fig. 3). In children with lower levels of self-esteem (> 0.93 *SD* below the mean), exploration was *higher* after modest praise than after inflated praise or no praise. By contrast, in children with higher levels of self-esteem (> 1.07 *SD* above the mean), exploration was *lower* after modest praise than after inflated praise or no praise.

### Robustness analyses

We examined the robustness of these results (Table S5, Supplementary Information). First, we reran the analyses without cases with a deviation from the experimental protocol. Specifically, we reran the analyses three times: once excluding cases where parents misworded the praise (e.g., when parents were instructed to give modest praise but inadvertently gave inflated praise; *n* = 12 cases), once excluding cases with other deviations from the experimental protocol (e.g., children reporting blurry vision in the VR task; *n* = 10 cases), and once excluding all aforementioned cases combined (*n* = 22 cases). Second, we reran the analyses without trials with undue influence on the parameter estimates (Cook’s distance > 1; *n* = 1 trial). Third, we performed model-based parametric bootstrapping (where we generated new values of both the random effects and their errors with parameters corresponding to the fitted model; *n* = 5000) and semi-parametric bootstrapping (where the random effects errors are sampled from the distribution of residuals) to derive more robust estimates, confidence intervals, and *p*-values. In all these cases, the results were robust: all significant effects remained significant, and no non-significant effect became significant.

Although children were aware that parents watched them perform all trials, it is possible that failure on the last trial before they received praise made the praise less credible to them and reduced their exploration. This was not the case, however, as there was no significant interaction between praise and feedback (success, failure) on the final pre-manipulation trial in predicting perceived sincerity, *p* = 0.662 (Table S6) or exploration, *ps* ≥ 0.360 (Table S7). This attests to the robustness of our findings.

### Exploratory analyses

Did children with lower levels of self-esteem explore more after modest praise simply because the praise felt more sincere, accurate, or warranted to them? We conducted three exploratory analyses to address this question.

First, we asked: Did children with lower levels of self-esteem rate their parent’s words as more sincere after receiving modest praise? An ANOVA examined the effects of praise and self-esteem on perceived sincerity. There was a significant main effect of praise, *F*(2, 186) = 3.60, *p* = 0.029, with children reporting higher perceived sincerity after modest praise (*M* = 2.89, *SD* = 0.77) than after no praise (*M* = 3.23, *SD* = 0.69), *p* = 0.022. There was no significant difference in perceived sincerity between inflated praise (*M* = 3.08, *SD* = 0.69) and modest praise, *p* = 0.281, or no praise, *p* = 0.474. There was no significant effect of self-esteem, *F*(1, 186) = 2.31, *p* = 0.130, nor a significant praise × self-esteem interaction, *F*(2,186) = 0.26, *p* = 0.770. This shows that children with lower levels of self-esteem were not significantly more inclined than others to perceive their parents as sincere after receiving modest praise.

Second, we asked: Was perceived sincerity related to the level of exploration in children with lower levels of self-esteem? We ran a mixed effects regression model with exploration during the 30 post-manipulation trials as the Level-1 dependent variable. We included pre-manipulation exploration, praise as two dummy coded variables (dummy 1: 1 = modest praise, 0 = inflated praise, − 1 = no praise; dummy 2: 1 = inflated praise, 0 = modest praise, − 1 = no praise), self-esteem (mean-centered), and perceived sincerity of praise (mean-centered), as well as the two-way and three-way interactions between all variables as Level-2 fixed effects. We set a random intercept per individual. Results mirrored those of main analyses (Table S8). Importantly, the main effect, the two-way interactions, and the three-way interactions involving perceived sincerity of praise were all non-significant, *ps* ≥ 0.203. This shows that perceived sincerity was not significantly related to the level of exploration, regardless of children’s level of self-esteem.

Third, we asked: Did children with lower levels of self-esteem perform more poorly on the task or have fewer successful trials? If so, they might explore more after modest praise because they perceive such praise (unlike inflated praise) as accurate or warranted. Spearman rho rank correlations showed no significant association between self-esteem and the median of pre- and post-manipulation error, *r*(190) = − 0.01, *p* = 0.932 and *r*(190) = − 0.03, *p* = 0.695, respectively. Similarly, Poisson regressions showed no significant association between self-esteem and the frequency of pre- and post-manipulation success feedback, *B* = 0.02, *SE* = 0.04, *p* = 0.622, and *B* = 0.001, *SE* = 0.04, *p* = 0.974, respectively. This shows that self-esteem was not significantly related to task performance or the number of successful trials.

## Discussion

How can parents encourage exploration in children? In some cases, well-intentioned actions can curb children’s exploration. For example, when an experimenter demonstrates the function of a toy, children are less likely to explore different ways to use the toy^8^. Extending this emerging line of research, we examined, for the first time, the effects of parental praise on children’s motor exploration. We involved parents as naïve experimenters to provide children with praise, and we tracked children’s exploration in a controlled virtual reality environment. We defined motor exploration as children’s movement variability following failure, which has recently become regarded as a critical ingredient for motor learning^4^. Relative to no praise, modest praise encouraged exploration in children with lower levels of self-esteem, making them adapt their movements in response to failure. By contrast, modest praise discouraged exploration in children with higher levels of self-esteem. Such effects were not found for inflated praise. Together, these findings provide the first experimental evidence that modest praise can both spark and curb children’s motor exploration, depending on children’s pre-existing self-esteem levels.

### Theoretical implications

Consistent with our hypotheses, children with lower levels of self-esteem explored more after receiving modest praise than after receiving no praise. Inflated praise did not have such exploration-enhancing effects. What psychological mechanism underlies these findings? Children with lower levels of self-esteem tend to doubt their ability^24^, fear that others will evaluate them negatively^44^, and avoid settings that may expose their weaknesses^25^. Such anxiety may lead them to display redundant, rigid, and repetitive behaviors^55^, which can reduce their exploration^10^. Consistent with this view, after receiving no praise, children with lower levels of self-esteem were marginally less inclined to explore. Unlike inflated praise, modest praise may alleviate children’s anxiety by setting an achievable standard for their future performance. When children are praised in modest ways, they may feel acknowledged and valued, without feeling pressured to excel^19,56^. Accordingly, modest praise may give them the reassurance they need to explore.

While modest praise encouraged exploration in children with lower levels of self-esteem, it discouraged exploration in children with higher levels of self-esteem. Although unexpected, this finding is consistent with an earlier study showing that modest praise can lead children with higher levels of self-esteem to avoid challenging tasks^19^. Children with higher levels of self-esteem are confident in their ability^24^ and are eager to showcase their ability^25^. When they receive modest praise, they may wonder why others do not perceive them as positively as they perceive themselves. This could lead to a state of anxiety that reduces exploration^10^. Future research should examine this possibility directly.

There are alternative explanations for why modest praise was more beneficial to children with lower levels of self-esteem than to other children. One is that children with lower levels of self-esteem are more used to receiving modest praise from their parents. Familiar statements are more likely to be accepted as true^57,58^, so if children with lower levels of self-esteem often receive modest praise from their parents in everyday life, they might be more likely to accept such praise as true, giving them the self-confidence they need to explore. Challenging this interpretation, research shows that children with lower levels of self-esteem tend to receive more inflated praise—not more modest praise—from their parents^19,20^. Another alternative explanation is that children with lower levels of self-esteem perceive modest praise as more accurate or warranted. Praise that is perceived to be accurate and warranted tends to be motivating^59,60^. However, in our study, self-esteem was unrelated to children’s successes and performance on the task, indicating that modest praise was not objectively more accurate or warranted for children with lower levels of self-esteem. Consistent with these findings, among children who received modest praise, self-esteem was unrelated to how sincere children thought their parents were.

Unlike modest praise, inflated praise did not impact children’s exploration. Why not? One perspective suggests that inflated praise conveys to children an implicit demand for excellent performance^19^, which could induce a state of anxiety that constrains exploration, especially in children with lower levels of self-esteem. This would be consistent with research in adults, which has shown that individuals with lower levels of self-esteem sometimes respond unfavorably to extremely positive evaluations (e.g., they feel unsure as to whether they will be able to live up to the praise)^61,62^. However, because children in our study performed a novel task and their performance was visible to observers, perhaps those with lower levels of self-esteem already felt quite anxious, leaving little room for inflated praise to raise their anxiety. To verify this possibility, future research should unobtrusively track children’s level of anxiety (e.g., heart rate variability, electrodermal activity) and examine to what extent it underlies exploration. Another perspective suggests that the association between positive feedback and motivation follows an inverted U-shape, such that praise that is too positive fails to enhance motivation^63,64^, thereby failing to spark motor exploration^65^.

### Understanding the social context of exploration

By uncovering the causal effects of parental praise on children’s motor exploration, our research extends the broader literature on how parents can encourage children’s exploration. While parents can encourage children’s exploration directly by engaging with children in the task (e.g., asking questions about the task^12,36^), they can also encourage exploration indirectly by creating a supportive environment. Most notably, past work has revealed that parents can encourage exploration by being responsive to children’s exploratory behaviors (e.g., responding promptly and contingently)^35,66^, supporting children’s autonomy (e.g., showing positive encouraging affect)^38^, and cultivating children’s sense of competence (e.g., praising children’s actions)^14^. For example, one correlational study^14^ has shown that mothers’ statements of praise and encouragement—such as “What a great job you are doing!”—predicted children’s attempts to complete a task independently, without help from the parent. Our findings extend this work by isolating the causal effects of praise, focusing on motor exploration, and showing that parental praise is not invariably beneficial to exploration.

Our finding that praise is not invariably beneficial to children is consistent with the larger literature on praise^7^. Over the past two decades, much research has examined the effects of person and process praise (for reviews, see refs ^16,56^). When children are praised for their personal qualities (person praise; e.g., “You’re so smart at this”), they often become focused on *demonstrating* their ability. When they struggle or fail, they may question their ability and enter a helpless mode^18,67,68^. Conversely, when children are praised for their efforts or strategies (process praise; e.g., “You found a good way to do it”), they often become focused on *developing* their ability. When they struggle or fail, they often do not question their ability and remain in a mastery-oriented mode^18,67,68^. In our study, we used process praise, and we varied whether the praise was modest or inflated. To date, the effects of person versus process praise on exploration have not been studied directly. It is possible, for example, that process praise is more effective in encouraging exploration than is person praise, as it focuses children on developing—rather than demonstrating—their ability. This might be especially true for process praise that is modest, as it does not pressure children to excel. Addressing these questions will deepen our understanding of how praise shapes children’s exploration.

### Strengths, limitations, and research directions

Our study has several methodological strengths, including its experimental design, its involvement of parents as naïve experimenters, and its controlled virtual reality environment to capture children’s exploration. Our study also has limitations, which point toward promising avenues for future work. First, our study focused on late childhood, when children are highly sensitive to adults’ evaluations of them^24,40^. How would praise impact exploration in 18-month-old infants, who are already sensitive to subtle linguistic cues in parental language^69^? And how would praise impact exploration in adolescents, who are more likely to reject, or even rebel against, adults’ evaluations of them^70^? Second, by wearing a virtual reality headset, children did not receive any social feedback other than praise. What are other types of social feedback through which parents can encourage exploration? For example, can parents encourage exploration by construing failure as something beneficial rather than harmful^71^, providing supportive touch when children are wary of exploration^72^, or showing excitement or surprise when they themselves explore^73^? Third, our study examined the effects of a single instance of praise. Although we found small yet robust effects, these effects may compound over time in children’s everyday lives. Indeed, in their everyday lives, children are praised frequently^20,68^, especially in Western society^74^. Would children gradually habituate to frequent praise or would they become increasingly sensitized to it? Fourth, our task was too brief to investigate whether exploration would lead to improvements in children’s performance over time. Exploration is crucial for motor learning as it allows children to find new task solutions^2^. If modest praise sparks exploration in children with lower levels of self-esteem, would it improve their performance in the long run? Addressing these questions will uncover the reach and impact of praise on children’s exploration.

## Conclusion

Western society has embraced the practice of praising children^75^. Self-help books state, “Be generous with your praise. Find as many opportunities to sincerely praise your children as you can”^76^. Our research shows that praise is not invariably beneficial. While inflated praise does not benefit children’s exploration, modest praise—a simple “You did well!”—can cultivate exploration in children with lower levels of self-esteem, leading them to adapt their actions to failure. Thus, when phrased modestly, praise can help children with lower levels of self-esteem embrace the gift of failure.

## Supplementary Information

Supplementary Information.

## Data Availability

The study data, code, questionnaires, and codebook are available on OSF at: https://osf.io/u3ma8/.
