# Supplementary material for: Parental praise and children’s exploration: a virtual reality experiment
Source: Sci Rep. 2022 Mar 23;12:4967. doi: 10.1038/s41598-022-08226-9 (PMC8943146; doi:10.1038/s41598-022-08226-9)
Supplement: Supplementary file 1 — Supplementary Information. [file 41598_2022_8226_MOESM1_ESM.docx]

**Supplementary Information**

**Parental Praise and Children’s Exploration: A Virtual Reality Experiment**

Eddie Brummelman, Stathis Grapsas, and Katinka van der Kooij

**Figure S1**

*Success Feedback, Failure Feedback, and Task Performance*


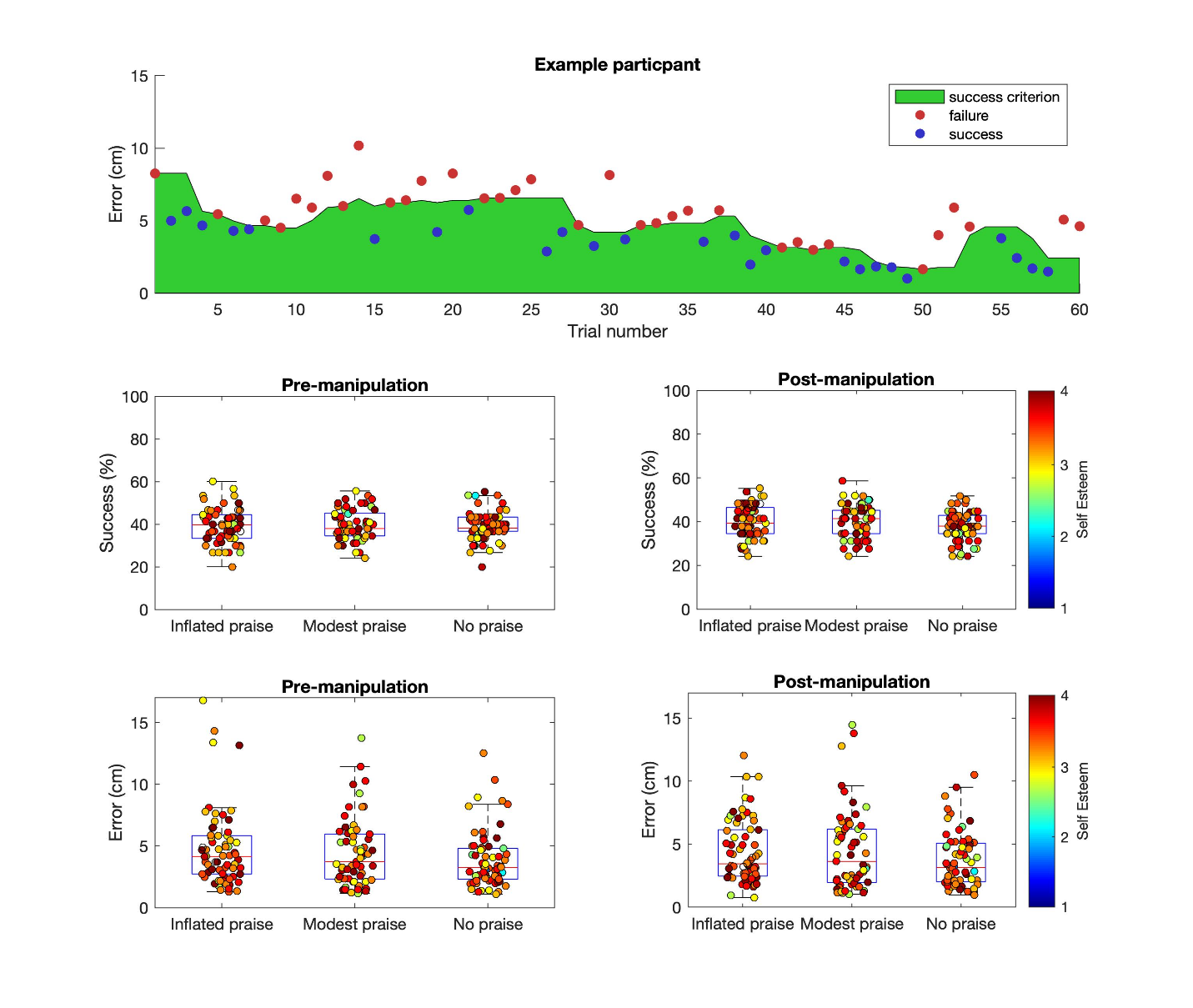


*Note.* The top panel shows how success feedback (blue dots) and failure feedback (red dots) was determined based on an adaptive success criterion, based on a participant’s performance relative to their own performance on previous trials. The middle panel shows the percentage of successful trials across conditions. The bottom panel shows the median error across conditions. Boxplots represent medians with confidence intervals, with color-coded dots representing different levels of self-esteem.

**Table S1**

*Number of Success and Failure Trials per Task Phase and Experimental Condition*

|  | | Success | | | Failure | | |
| --- | --- | --- | --- | --- | --- | --- | --- |
|  | | Range | *M* | *SD* | Range | *M* | *SD* |
| Pre manipulation | |  |  |  |  |  |  |
|  | Modest praise | 7-16 | 11.67 | 2.06 | 14-23 | 18.33 | 2.06 |
|  | Inflated praise | 6-18 | 11.57 | 2.35 | 12-24 | 18.43 | 2.35 |
|  | No praise | 6-16 | 11.55 | 1.92 | 14-24 | 18.45 | 1.92 |
|  | Across conditions | 6-18 | 11.59 | 2.11 | 12-24 | 18.41 | 2.11 |
| Post manipulation | |  |  |  |  |  |  |
|  | Modest praise | 7-17 | 11.41 | 2.08 | 12-22 | 17.59 | 2.08 |
|  | Inflated praise | 7-16 | 11.48 | 2.02 | 13-22 | 17.52 | 2.02 |
|  | No praise | 7-15 | 10.86 | 1.90 | 14-22 | 18.14 | 1.90 |
|  | Across conditions | 7-17 | 11.24 | 2.01 | 12-22 | 17.76 | 2.01 |
| Full task | |  |  |  |  |  |  |
|  | Modest praise | 18-32 | 23.08 | 2.65 | 27-41 | 35.92 | 2.65 |
|  | Inflated praise | 17-29 | 23.05 | 2.56 | 30-42 | 35.95 | 2.56 |
|  | No praise | 15-28 | 22.41 | 2.65 | 31-44 | 36.59 | 2.65 |
|  | Across conditions | 15-32 | 22.84 | 2.63 | 27-44 | 36.16 | 2.63 |

*Note.* This table is based on our final sample (*N* = 192), prior to exclusion of trials with movement artifacts. Participants completed 60 trials. The numbers of successes and failures add up to 59 per participant, because feedback on the final trial was not analyzed, as there was no subsequent trial to index movement variability. The task was programmed to give all children failure feedback on the first trial but, due to a technical error, seven children received success feedback on this trial**.**

**Table S2**

*Average Movement Variability in Centimeters per Task Phase and Experimental Condition*

|  |  | Post-success trials | | Post-failure trials | | All trials | |
| --- | --- | --- | --- | --- | --- | --- | --- |
|  |  | *M* | *SD* | *M* | *SD* | *M* | *SD* |
| Pre manipulation | |  |  |  |  |  |  |
|  | Modest praise | 2.32 | 1.77 | 3.24 | 3.16 | 2.88 | 2.73 |
|  | Inflated praise | 2.36 | 1.75 | 3.00 | 2.51 | 2.75 | 2.26 |
|  | No praise | 2.61 | 2.64 | 3.25 | 4.35 | 3.00 | 3.78 |
|  | Across conditions | 2.43 | 2.11 | 3.16 | 3.45 | 2.88 | 3.02 |
| Post manipulation | |  |  |  |  |  |  |
|  | Modest praise | 2.16 | 1.47 | 2.78 | 2.09 | 2.54 | 1.89 |
|  | Inflated praise | 2.23 | 1.37 | 2.72 | 1.72 | 2.52 | 1.61 |
|  | No praise | 2.37 | 2.17 | 2.87 | 2.32 | 2.68 | 2.28 |
|  | Across conditions | 2.26 | 1.71 | 2.79 | 2.06 | 2.58 | 1.95 |
| Full task | |  |  |  |  |  |  |
|  | Modest praise | 2.24 | 1.63 | 3.01 | 2.69 | 2.71 | 2.36 |
|  | Inflated praise | 2.30 | 1.57 | 2.69 | 2.16 | 2.64 | 2.64 |
|  | No praise | 2.49 | 2.43 | 3.06 | 3.49 | 2.84 | 3.14 |
|  | Across conditions | 2.35 | 1.92 | 2.98 | 2.85 | 2.73 | 2.55 |

*Note.* This table is based on our final sample (*N* = 192), after exclusion of trials with movement artifacts.

**Table S3**

*Multilevel Analysis Examining the Effects of Age, Praise and Self-Esteem on Children’s Exploration*

| Fixed Effects | *B* | *SE (B)* | *t* |
| --- | --- | --- | --- |
| Intercept | 1.58 | 0.48 | 3.27*** |
| Pre-manipulation exploration | 0.45 | 0.03 | 15.12*** |
| Age | -0.02 | 0.05 | -0.52 |
| Modest Praise^a^ | 0.00 | 0.08 | 0.02 |
| Inflated Praise^b^ | -0.03 | 0.08 | -0.44 |
| Self-Esteem | -0.01 | 0.10 | -0.14 |
| Modest Praise × Self-Esteem | -0.37 | 0.15 | -2.51* |
| Inflated Praise × Self-Esteem | 0.10 | 0.16 | 0.62 |

^a^Modest Praise: 1 = Modest Praise, 0 = Inflated Praise, -1 = No Praise.

^b^Inflated praise: 1 = Inflated Praise, 0 = Modest Praise, -1 = No Praise.

**p* < .05. ***p* < .01. ****p* < .001.

**Table S4**

*Awareness Check: The Feedback That Children Reported to Have Received, per Experimental Condition*

|  |  |  | | Children’s response to “What did your parent tell you?” | | | | | |
| --- | --- | --- | --- | --- | --- | --- | --- | --- | --- |
|  |  | *n* | | “You did well!” | | “You did *incredibly* well!” | | neither | |
|  |  | *Full Sample* | *Analyses Sample* | *Full Sample* | *Analyses Sample* | *Full Sample* | *Analyses Sample* | *Full Sample* | *Analyses Sample* |
|  | Modest praise | 64 | 61 | 40 | 37 | 10 | 10 | 13 | 13 |
| Condition | Inflated praise | 68 | 65 | 15 | 15 | 41 | 39 | 10 | 10 |
|  | No praise | 68 | 66 | 25 | 24 | 4 | 4 | 38 | 38 |

**Table S5**

*Results of Robustness Tests for Main Analyses*

|  | *Robustness Test 1* | | |  | *Robustness Test 2* | | |  | *Robustness Test 3* | | |  | *Robustness Test 4* | | |  | *Robustness Test 5* | | | | | | | | |
| --- | --- | --- | --- | --- | --- | --- | --- | --- | --- | --- | --- | --- | --- | --- | --- | --- | --- | --- | --- | --- | --- | --- | --- | --- | --- |
|  | Exclusion of Cases With Misworded Praise  (*n* = 12) | | |  | Exclusion of Cases With Other Protocol Deviations (*n* = 10) | | |  | Exclusion of All Cases With Protocol Deviations (*n* = 22) | | |  | Exclusion of Trials With Cook’s D > 1 (*n* = 1) | | |  |  |  | Parametric Model-Based Bootstrap | | |  | Semi-Parametric Model-Based Bootstrap | | |
| Fixed Effects | *B* | *SE* | *t* |  | *B* | *SE* | *t* |  | *B* | *SE* | *t* |  | *B* | *SE* | *t* |  | Original *B* |  | Bias | *SE* | 95% CI |  | Bias | *SE* | 95% CI |
| Intercept | 1.35 | 0.11 | 12.01*** |  | 1.34 | 0.11 | 12.00*** |  | 1.35 | 0.12 | 11.54*** |  | 1.49 | 0.11 | 13.22*** |  | 1.33 |  | -0.0006 | 0.11 | (1.122, 1.546) *** |  | -0.0004 | 0.06 | (1.21, 1.45) *** |
| Pre-manipulation exploration | 0.45 | 0.03 | 14.97*** |  | 0.45 | 0.03 | 15.36*** |  | 0.45 | 0.03 | 14.47*** |  | 0.40 | 0.03 | 13.35*** |  | 0.45 |  | -0.0001 | 0.03 | (0.40, 0.51) *** |  | 0.0002 | 0.02 | (0.42, 0.49) *** |
| Modest Praise^a^ | -0.02 | 0.09 | -0.20 |  | 0.01 | 0.08 | 0.09 |  | 0.00 | 0.09 | -0.05 |  | 0.01 | 0.08 | 0.12 |  | 0.00 |  | 0.0012 | 0.08 | (-0.16, 0.16) |  | -0.0042 | 0.05 | (-0.10, 0.09) |
| Inflated Praise^b^ | -0.04 | 0.08 | -0.44 |  | -0.05 | 0.08 | -0.60 |  | -0.05 | 0.08 | -0.62 |  | -0.02 | 0.08 | -0.3 |  | -0.03 |  | -0.0003 | 0.08 | (-0.19, 0.12) |  | 0.0064 | 0.04 | (-0.11, 0.06) |
| Self-Esteem | -0.02 | 0.11 | -0.22 |  | -0.01 | 0.11 | -0.12 |  | -0.02 | 0.11 | -0.17 |  | 0.01 | 0.11 | 0.13 |  | -0.02 |  | 0.0000 | 0.1 | (-0.21, 0.18) |  | 0.0019 | 0.06 | (-0.13, 0.09) |
| Modest Praise × Self-Esteem | -0.42 | 0.16 | -2.62** |  | -0.38 | 0.15 | -2.50* |  | -0.42 | 0.16 | -2.59* |  | -0.42 | 0.15 | -2.75** |  | -0.37 |  | -0.0036 | 0.14 | (-0.65, -0.09)* |  | -0.0039 | 0.08 | (-0.54, -0.21) *** |
| Inflated Praise × Self-Esteem | 0.11 | 0.16 | 0.66 |  | 0.10 | 0.16 | 0.62 |  | 0.11 | 0.17 | 0.64 |  | 0.08 | 0.16 | 0.47 |  | 0.10 |  | 0.001 | 0.16 | (-0.21, 0.41) |  | 0.0064 | 0.09 | (-0.07, 0.27) |

^a^Modest Praise: 1 = Modest Praise, 0 = Inflated Praise, -1 = No Praise.

^b^Inflated praise: 1 = Inflated Praise, 0 = Modest Praise, -1 = No Praise.

**p* < .05. ***p* < .01. ****p* < .001.

**Table S6**

*ANOVA Predicting Perceived Sincerity Based on Experimental Condition, Feedback on the Final Pre-Manipulation Trial, and Their Interaction*

|  | *df* | Sum of Squares | Mean of Squares | *F* | *Sig.* |
| --- | --- | --- | --- | --- | --- |
| 30^th^ Trial Feedback | 1 | 0.00 | 0.00 | 0.00 | .981 |
| Condition | 2 | 3.72 | 1.86 | 3.57 | .030 |
| 30^th^ Trial Feedback × Condition | 2 | 0.43 | 0.22 | 0.41 | .662 |
| Residuals | 186 | 97.12 | 0.52 | - | - |

*Note.* Condition = Experimental condition (modest praise, inflated praise, no praise). 30^th^ Trial Feedback = Success (vs Failure) feedback after the 30^th^ trial

**Table S7**

*Multilevel Analysis Examining the Effects of Feedback Type on the Last Pre-manipulation Trial, Praise, and Self-Esteem on Children’s Exploration*

| Fixed Effects | *B* | *SE (B)* | *t* |
| --- | --- | --- | --- |
| Intercept | 1.30 | 0.12 | 11.029*** |
| Pre-manipulation exploration | 0.45 | 0.03 | 15.693*** |
| 30^th^ Trial Feedback^a^ | 0.06 | 0.11 | 0.51 |
| Modest Praise^b^ | -0.06 | 0.11 | -0.57 |
| Inflated Praise^c^ | 0.03 | 0.10 | 0.27 |
| Self-Esteem | -0.02 | 0.10 | -0.21 |
| 30^th^ Trial Feedback × Modest Praise | 0.13 | 0.16 | 0.81 |
| 30^th^ Trial Feedback × Inflated Praise | -0.15 | 0.16 | -0.92 |
| Modest Praise × Self-Esteem | -0.38 | 0.15 | -2.59* |
| Inflated Praise × Self-Esteem | 0.10 | 0.16 | 0.64 |

^a^30^th^ Trial Feedback: 1 = Success, 0 = Failure.

^b^Modest Praise: 1 = Modest Praise, 0 = Inflated Praise, -1 = No Praise.

^c^Inflated praise: 1 = Inflated Praise, 0 = Modest Praise, -1 = No Praise.

**p* < .05. ***p* < .01. ****p* < .001.

**Table S8**

*Multilevel Analysis Examining the Effects of Praise, Self-Esteem, and Perceived Sincerity on Children’s Exploration*

| Fixed Effects | *B* | *SE (B)* | *t* |
| --- | --- | --- | --- |
| Intercept | 1.25 | 0.12 | 10.75*** |
| Pre-manipulation exploration | 0.48 | 0.03 | 14.88*** |
| Modest Praise^a^ | -0.03 | 0.08 | -0.35 |
| Inflated Praise^b^ | -0.03 | 0.08 | -0.36 |
| Self-Esteem | 0.01 | 0.11 | 0.14 |
| Perceived Sincerity | -0.05 | 0.08 | -0.70 |
| Modest Praise × Self-Esteem | -0.36 | 0.15 | -2.38* |
| Inflated Praise × Self-Esteem | 0.12 | 0.16 | 0.74 |
| Modest Praise × Perceived Sincerity | -0.09 | 0.11 | -0.83 |
| Inflated Praise × Perceived Sincerity | -0.06 | 0.11 | -0.50 |
| Self-Esteem × Perceived Sincerity | -0.20 | 0.15 | -1.30 |
| Modest Praise × Self-Esteem × Perceived Sincerity | 0.29 | 0.22 | 1.28 |
| Inflated Praise × Self-Esteem × Perceived Sincerity | -0.13 | 0.23 | -0.56 |

^a^Modest Praise: 1 = Modest Praise, 0 = Inflated Praise, -1 = No Praise.

^b^Inflated praise: 1 = Inflated Praise, 0 = Modest Praise, -1 = No Praise.

**p* < .05. ***p* < .01. ****p* < .001.
